# CXCR4‐SF1 bifunctional adipose‐derived stem cells benefit for the treatment of Leydig cell dysfunction‐related diseases

**DOI:** 10.1111/jcmm.15128

**Published:** 2020-03-17

**Authors:** Xue Li, Ao Xu, Kai Li, Jie Zhang, Qin Li, Gang Zhao, Yue Zhang, Hang Yuan, Yafei Guo, Ping Lin, Lugang Huang

**Affiliations:** ^1^ Department of Pediatric Surgery West China Hospital Sichuan University Chengdu China; ^2^ Lab of Experimental Oncology State Key Laboratory of Biotherapy and Cancer Center National Clinical Research Center for Geriatrics West China Hospital of Sichuan University Chengdu China

**Keywords:** adipose‐derived stem cell, CXCR4, SF1, transplantation

## Abstract

Stem cell transplantation is a candidate method for the treatment of Leydig cell dysfunction‐related diseases. However, there are still many problems that limit its clinical application. Here, we report the establishment of CXCR4‐SF1 bifunctional adipose‐derived stem cells (CXCR4‐SF1‐ADSCs) and their reparative effect on Leydig cell dysfunction. CD29^+^ CD44^+^ CD34^−^ CD45^−^ ADSCs were isolated from adipose tissue and purified by fluorescence‐activated cell sorting (FACS). Infection with lentiviruses carrying the CXCR4 and SF1 genes was applied to construct CXCR4‐SF1‐ADSCs. The CXCR4‐SF1‐ADSCs exhibited enhanced migration and had the ability to differentiate into Leydig‐like cells in vitro. Furthermore, the bifunctional ADSCs were injected into BPA‐mediated Leydig cell damage model mice via the tail vein. We found that the CXCR4‐SF1‐ADSCs were capable of homing to the injured testes, differentiating into Leydig‐like cells and repairing the deficiency in reproductive function caused by Leydig cell dysfunction. Moreover, we investigated the mechanism underlying SF1‐mediated differentiation and testosterone synthesis in Leydig cells, and the B‐box and SPRY Domain Containing Protein (BSPRY) gene was proposed to be involved in this process. This study provides insight into the treatment of Leydig cell dysfunction‐related diseases.

## 1 INTRODUCTION

Leydig cells, which synthesize and secrete testosterone, are critical to the development and maintenance of the normal functions of the male reproductive system.^1^, ^2^ Various diseases (such as cryptorchidism) and environmental factors can lead to Leydig cell dysfunction, resulting in androgen deficiency‐related diseases. Studies have shown that environmental oestrogen analogs (such as bisphenol A (BPA), 2,2‐bis(4‐hydroxyphenyl) propane) can reduce the spermatogenic ability by damaging Leydig cells and inhibiting testosterone secretion.^3^ We previously found that obesity could lead to decreases in the number of Leydig cells, testosterone secretion and reproductive function in mice.^4^

Currently, hormone replacement therapy is used as a clinical treatment for these diseases, but it is limited by its inability to control the hypothalamus‐pituitary axis and its side effects.^5^, ^6^, ^7^ In addition, stem cell techniques show great promise in the treatment of Leydig cell dysfunction, and some preclinical trials have been conducted. It may offer a non‐hormonal, potentially permanent option that would be within a working hormonal axis. Some studies have indicated that Leydig stem cells isolated from the testes can be implanted into Leydig cell‐injured testes to increase the testosterone level.^8^, ^9^ Besides, this method could not only provide therapeutic benefits for men who would otherwise not have treatment options (such as Klinefelters), but also avoid the issues associated with excessive or insufficient hormone supplementation. However, because of difficulties in sourcing Leydig stem cells and ethical concerns, the popularization and application of this approach in the clinic are limited. Therefore, methods to effectively reduce the occurrence of androgen deficiency diseases, improve reproductive system development and dysfunction caused by hypoandrogenism and treat patients effectively have become urgently needed in society.

Mesenchymal stem cells (MSCs) have multilineage differentiation potential and greater potential for application than Leydig stem cells. Compared with other MSCs, adipose‐derived stem cells (ADSCs) have the advantages of easy acquisition, reduced immunogenicity and an enhanced differentiation capacity.^10^, ^11^, ^12^ Many preclinical studies have studied the roles of ADSCs in promoting chronic diabetic wound healing and skin regeneration as well as the regeneration of cartilage tissue and liver cells.^13^, ^14^, ^15^, ^16^ ADSC therapy for Leydig cell dysfunction‐related diseases has also been evaluated. Yang et al^17^ injected ADSCs into an ageing rat model and found that these ADSCs migrated to damaged testis areas and the testosterone level increased. However, they noted that the effect of the ADSCs was more likely to be the result of reduced apoptosis than direct differentiation. Furthermore, we successfully induced ADSCs to differentiate into Leydig‐like cells, and the expression of 3β‐HSD and testosterone was previously detected in the induced cells.^18^ All these findings indicate that ADSCs have great potential and value in the treatment of Leydig cell dysfunction‐related diseases.

It is known that the self‐renewal and differentiation of stem cells depend on the functional ecological pool formed by the cells and the cytokines they secrete.^19^, ^20^ The differentiation of Leydig cells in vivo depends on the testicular microenvironment. Therefore, there are two problems in repairing Leydig cell dysfunction with ADSCs: one is how to effectively induce ADSCs to migrate to the sites of Leydig cell dysfunction in the testes so that these cells can be regulated by the testicular microenvironment, and the other is how to improve the ability of ADSCs to differentiate into Leydig‐like cells after colonization of the testes. Zhiyv et al^21^ reported that the stromal cell‐derived factor‐1 (SDF‐1)/CXC chemokine receptor‐4 (CXCR4) pathway is the most important biological axis for promoting MSC homing to injured tissues and that MSCs positive for CXCR4 expression can migrate to injured tissues with the help of SDF‐1. However, in vitro MSC culture reduces CXCR4 expression and weakens this homing ability. Steroidogenic factor‐1 (SF1), also known as Ad4BP or NR5A1, is vital to the differentiation and development of the adrenal gland, gonads and pituitary gland and the synthesis of steroid hormones. Yazava et al^22^ found that bone marrow‐derived mesenchymal stem cells (BMSCs) transfected with SF1 could differentiate into Leydig‐like cells in vitro and that the expression of P450scc, 3β‐HSD and 17β‐HSD was detectable.

In this study, we constructed CXCR4‐SF1 bifunctional ADSCs (CXCR4‐SF1‐ADSCs) and injected them into model mice with BPA‐mediated Leydig cell injury via the tail vein. We found that these cells had the ability to home to the testes with Leydig cell dysfunction and differentiate into Leydig‐like cells in the testicular microenvironment. Furthermore, the mechanism of ADSC differentiation into Leydig‐like cells was preliminarily studied.

## 2 MATERIALS AND METHODS

### 2.1 Establishment of BPA‐induced Leydig cell damage model

Six‐week‐old Balb/c male mice were purchased from the DaShuo Experimental Animal Centre (Chengdu, China). Mice were divided into two groups randomly and then injected intraperitoneally with vehicle (corn oil, Sigma) or BPA (100 mg/kg, Sigma) for 5 days. The bodyweight and food intake of the mice were weighed every day. All mice were housed in a controlled condition of 12‐hour light/dark cycle at 22°C. All animal studies were performed in accordance with the relevant guidelines and regulations and approved by the Ethics Committee of West China Hospital, Sichuan University.

### 2.2 Isolation and characterization of mouse ADSCs

The groin adipose tissue was removed from the male mouse (6‐week‐old) and minced into pieces aseptically. Then, the minced tissue was digested with 0.25% trypsin (Gibco) at 37°C for 1 hour and followed by 1 mg/mL type I collagenase (Invitrogen) in Dulbecco's modified Eagle's medium (DMEM)/F12 (Invitrogen) at 37°C for 1 hour. The digest was treated with the red blood cell lysis buffer for 5 minutes and centrifuged at 240 *g* for 10 minutes at room temperature. The sample was washed with phosphate‐buffered saline (PBS) twice, filtered through a cell strainer at the size of 40‐µm pore (BD Falcon), resuspended with Balb/c mouse adipose‐derived mesenchymal stem cell complete medium (Cyagen) and cultured at 37°C under an atmosphere of 95% humidified air with 5% CO_2_.

The isolated ADSCs were characterized and sorted by flow cytometry with antibodies against the surface marker CD29, CD44, CD34 and CD45 (CD29‐APC, CD34‐FITC, CD44‐PE‐Cyanine7, CD45‐PE, eBioscience™). The ADSCs we got were positive for CD29 and CD44, while negative for CD34 and CD45.

### 2.3 Lentiviral infection and transplantation of ADSCs

Lentiviruses (pLV[Exp]‐EGFP:Puro‐EF1A) expressing SF1 (LV‐SF1) or CXCR4 (LV‐CXCR4) were ordered from GenePharma, China. A lentivirus (pLV[Exp]‐EGFP:T2A:Puro‐EF1A) that expressed CXCR4 and SF1 together (LV‐CXCR4‐SF1) was purchased from Cyagen, China. All lentiviruses contained the GFP gene and puromycin resistance gene.

Sorted ADSCs (2nd passage) in the logarithmic growth phase were placed in a 6‐well plate and incubated at 37°C under an atmosphere of 95% humidified air with 5% CO_2_ until the cell density reached 50% or 60%. Control and target gene lentiviruses (LV‐Vector, LV‐CXCR4, LV‐SF1 and LV‐CXCR4‐SF1) were placed on ice to melt, and the lentiviruses (MOI: 50) were diluted with 1 mL culture medium containing 10% foetal bovine serum and polybrene (5 µg/mL). Then, the mixture was added to the corresponding well after gentle mixing. The next day, the original medium was replaced with 2 mL fresh medium. Forty‐eight hours later, the fluorescence produced by the expression of GFP was observed with a fluorescence microscope. Puromycin (5 μg/mL, Solarbio Life Science) was applied to select and enrich for antibiotic‐resistant transfected cells. Thus, Vector‐ADSCs, CXCR4‐ADSCs, SF1‐ADSCs and CXCR4‐SF1‐ADSCs were established.

Each type of ADSCs (3 × 10^6^) was suspended in 0.1 mL sterile PBS and injected into vehicle‐ or BPA‐treated mice. Thus, we obtained 8 animal groups in this study, namely Vehicle‐Vector‐ADSCs, Vehicle‐CXCR4‐ADSCs, Vehicle‐SF1‐ADSCs, Vehicle‐CXCR4‐SF1‐ADSCs, BPA‐Vector‐ADSCs, BPA‐CXCR4‐ADSCs, BPA‐SF1‐ADSCs and BPA‐CXCR4‐SF1‐ADSCs.

### 2.4 Quantitative real‐time polymerase chain reaction (qRT‐PCR)

The total RNA was extracted from cells using RNAiso Plus (TAKARA), and reverse transcription reactions were performed by using a PrimeScript RT reagent kit (TAKARA) according to the manufacturer's instructions. qRT‐PCR was performed with SYBR Green Master Mix (TAKARA) and an iCycler iQTM Multicolour Real‐Time Detection System (BIO‐RAD). The information of primers was listed as follows:

**Table:** 

Genes	Forward (5′‐3′)	Reverse (3′‐5′)
18S	TTGACGGAAGGGCACCACCAG	GCACCACCACCCACGGAATCG
3β‐HSD	TGGACAAAGTATTCCGACCAGA	GGCACACTTGCTTGAACACAG
17β‐HSD	ATTCCACGAAGTGTACTGTGC	AGGGCTTGCTCATAACCACG
P450scc	AGGTCCTTCAATGAGATCCCTT	TCCCTGTAAATGGGGCCATAC
CYP17a1	GTCGCCTTTGCGGATAGTAGT	TGAGTTGGCTTCCTGACATATCA
StAR	CGGGTGGATGGGTCAAGTTC	GCACTTCGTCCCCGTTCTC
BSPRY	CACAATGGGTATCATGAGCCTCTG	CCTAGAAGCATGGCCTGACCTG

### 2.5 Western blotting analysis

The testes or collected cells were lysed with appropriate amount of lysis buffer containing RIPA buffer and protease inhibitor, and lysed on ice for 30 minutes. Then, the lysates were centrifuged (4°C, 24 000 *g*) and the supernatant was harvested. The protein content was detected in accordance with the Bio‐Rad Protein Assay (BIO‐RAD). Equivalent proteins extracted were loaded, separated via 12% SDS‐PAGE and transferred to a polyvinylidene difluoride membrane (Millipore). The membrane was blocked with TBST (10 mmol/L Tris‐HCl, pH 8.0, 150 mmol/L NaCl, 0.1% Tween‐20) containing 5% skim milk for 1 hour at 37°C, after that, the membrane was incubated with primary antibody at 4°C overnight. The anti‐StAR (D‐2) (sc‐166821) antibody, anti‐3β‐HSD (P‐18) (sc‐30820) antibody and anti‐H3 antibody were purchased from Santa Cruz Biotechnology. The anti‐BSPRY (bs‐12639R) and the anti‐SDF‐1 (bs‐4938R) antibodies were purchased from Bioss Biotechnology. After the incubation with primary antibody, the membrane was washed with TBST for three times and incubated with a horseradish peroxidase (HRP)‐conjugated goat anti‐rabbit (Sangon Biotech) for 1 hour at 37°C. And finally, the membrane was developed using Immobilon Western Chemiluminescent HRP Substrate (Millipore). Densitometric ratio analyses were performed using ImageJ software.

### 2.6 In vitro differentiation of Lentiviral‐infected ADSCs

The Vector‐ADSCs, CXCR4‐ADSCs, SF1‐ADSCs and CXCR4‐SF1‐ADSCs were induced into adipogenic and osteogenic differentiation to verify the multidirectional differentiation potential in vitro. The Balb/c Mouse Adipose‐derived Stem Cell Adipogenic Differentiation Medium (MUCMD‐90031) and the Balb/c Mouse Adipose‐derived Stem Cell Osteogenic Differentiation Medium (MUCMD‐90021) were purchased from Cyagen, and the induced procedure was conducted according to the instructions. After the inducement, Oil red O was used for adipogenesis identification, and Alizarin Red S was used for osteogenesis identification.

### 2.7 ELISA

The blood of the mouse was placed at 37°C for 3 hours and followed by 4°C for 1 hour. Next, the blood sample was centrifuged at 1500 *g* for 10 minutes at 4°C to get the serum. For testosterone measurement, the cell culture suspensions or the serum was collected and measured using a Testosterone ELISA Kit (ENZO, ADI‐900‐065) as the manufacturer's instructions.

### 2.8 Tissue preparation

The mouse was anaesthetized by intraperitoneal injection of chloral hydrate (10%) and killed by cervical dislocation. Immediately, the testes, epididymides, lung, kidney and liver were collected. Then, one side of the testes and epididymides was frozen in liquid nitrogen, while the other side was fixed mDF for 72 hours as reference.^23^, ^24^ The lung, kidney and liver were fixed in 4% paraformaldehyde for 48 hours.

To get the testis homogenates, the testis tissue frozen in liquid nitrogen was weighed, placed in normal saline (NS) containing protease inhibitor (a ratio of 0.1 g:1 mL) and homogenized on ice. After homogenization, the homogenate was centrifugation at 2800 *g* at 4°C for 15 minutes. The supernatant was collected and stored at −80°C.

### 2.9 Haematoxylin‐eosin (HE) staining, immunohistochemistry (IHC) and immunofluorescence (IF)

The tissues were paraffin embedded and then sliced into 4‐μm sections. Then, the sections were got dewaxed and hydrated, and stained by haematoxylin‐eosin (Beyotime Biotechnology) as the instructions. The Photo Imaging System (Olympus DP20) was applied to take the photomicrographs of these sections.

For IHC assay, 3% H_2_O_2_ was used to block the endogenous peroxidase for 15 minutes; then, high‐pressure antigen repair method was required to get the antigen retrieved. The primary antibodies were incubated overnight at 4°C. After that, these sections were incubated with horseradish peroxidase (HRP)‐conjugated goat anti‐rabbit antibodies (Sangon Biotech) (1:1000 dilution) for 1 hour at 37°C. Subsequently, the sections were counterstained with haematoxylin. Photomicrographs were also taken by a Photo Imaging System (Olympus DP20).

For IF assay, the work procedures were the same as the IHC assay until the application of the primary antibodies. Next, these sections were incubated with an Alexa Fluor 594‐conjugated secondary antibody (1:100 dilution; Boaosen) for 1 hour at 37°C. And DAPI (Invitrogen) was used for nuclei staining. The photomicrographs were taken by a fluorescence microscope (Olympus Optical Co).

### 2.10 Mating assay

The mating experiment was conducted 1 week, 3 weeks and 5 weeks after the cell injection. One male mouse was mated with two randomly selected normal female mice, and every group contained 5 male mice. Thus, these female mice got grouped the same with their male counterparts. The female mouse was separated to another cage after pregnancy. The numbers of pups were counted soon after the birth. At last, the average offspring number of every female mouse was counted.

### 2.11 Cell proliferation assay

Cell suspension (100 μL/well) was seeded into a 96‐well plate at a density of 3000 cells/well. The cell proliferation ability was detected by CCK8 kits (4A Biotech Co. Ltd) according to the instructions. All the experiments were repeated 3 times.

### 2.12 Transwell migration assay

3 × 10^4^ cells of the four lentivirus‐treated cells were separately seeded into the upper Transwell chambers at the size of 8 µm pore (Corning). Then, the upper chambers were placed into the 24‐well plate. Next, 500 μL normal culture medium containing FBS was placed in the lower chambers. The 24‐well plate was incubated at 37°C under an atmosphere of 95% humidified air with 5% CO_2_ for 48 hours. Finally, the upper chambers were fixed in 4% paraformaldehyde for 15 minutes and stained with 0.05% crystal violet for 15 minutes. Pictures were taken by a Photo Imaging System (Olympus DP20).

### 2.13 Analysis of sperm concentration, sperm motility and abnormal sperm rate

One side of the epididymides was put in a plate containing normal saline (preheated at 37°C) and cut into small pieces.^25^ The plate was gently mixed and placed in a 37°C water bath for 15 minutes. After that, the suspension was filtered with a cell filter. Then, the suspension was dropped on a glass slide to count the sperm concentration and observe the movement of the sperm by a microscope. Each suspension should be counted for 3 times. After the count of sperm concentration, 300 sperms of each mouse were included to calculate the motility index as following: perm motility index (%) = Number of motive sperms/number of examined sperms × 100%. The suspension was dropped on a glass slide and dried naturally. And the glass was fixed by 4% paraformaldehyde for 5 minutes. Finally, the sperms were stained by haematoxylin‐eosin and observed under a microscope (Olympus DP20). Abnormal sperm rate (%) = Number of abnormal sperms/number of examined sperms × 100%.

### 2.14 Statistical analysis

Data were presented as the means ± standard error of the mean (SEM). And SPSS software (version 20.0; SPSS, Inc, Chicago, IL) was used to perform statistical tests. The significance between groups was performed using Student's *t* test or unpaired *t* test with Welch's correction. One‐way analysis of variance (ANOVA), followed by Tukey's post hoc test was used among three or more groups. *P* < .05 was considered to indicate statistically significant.

## 3 RESULTS

### 3.1 Construction of CXCR4‐SF1‐ADSCs

**Figure 1:**
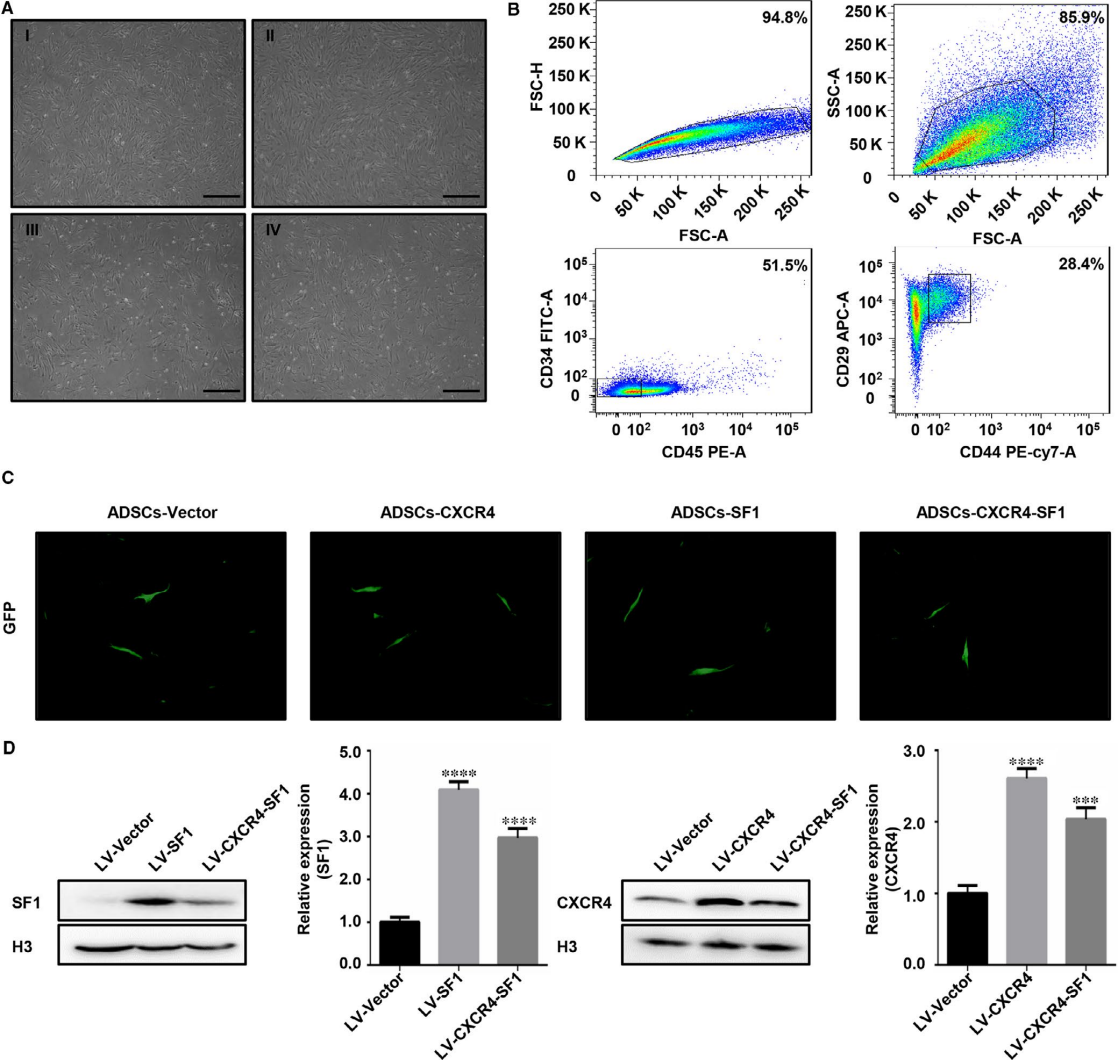
The isolation, characterization and sorting of mouse adipose‐derived stem cells (ADSCs). (A) The morphology of isolated mouse ADSCs. (I) P0, the primary cells isolated from adipose tissue; (II) P1, passage 1, cells sorted by flow cytometry; (III) P2, passage 2; and (IV) P3, passage 3. Scale bar, 100 µm. (B) Flow cytometry characterization and sorting of the ADSCs. (C) GFP (green) expression in sorted ADSCs infected with a lentivirus (LV‐Vector, LV‐CXCR4, LV‐SF1 or LV‐CXCR4‐SF1). Scale bar, 25 µm. (D) The protein and mRNA levels of CXCR4 and SF1 in lentivirus‐infected ADSCs

Adipose‐derived stem cells were isolated from mouse inguinal adipose tissue by enzymatic digestion. Two days after inoculation into a cell culture dish, the cells began to grow adherently on the dish. Most of the cells exhibited a spindle‐shaped morphology (Figure 1A I). The cells were evaluated and selected by FACS according to the expression of molecular surface markers of mouse adipose stem cells. Approximately 28.4% of the cells were CD29^+^ CD44^+^ CD34^−^ CD45^−^, and they were defined as ADSCs according to previous studies^26^, ^27^, ^28^ (Figure 1B). The ADSCs we sorted were cultured and used for subsequent experiments. After three passages, the ADSCs retained the spindle‐shaped morphology (Figure 1A II, III and IV).

Next, we used lentiviral infection to construct different types of ADSCs, which were named Vector‐ADSCs, CXCR4‐ADSCs, SF1‐ADSCs and CXCR4‐SF1‐ADSCs; the Vector‐ADSCs were used as a negative control. All lentiviruses carried a GFP gene, and green fluorescence could be detected under a fluorescence microscope for these four types of ADSCs (Figure 1C). Subsequently, Western blotting and qPCR were used to detect the expression of CXCR4 and SF1 at the protein and mRNA levels (Figure 1D). These results indicated that the lentivirus‐infected ADSCs were successfully established. Whether a lentivirus can affect the pluripotency of ADSCs has not been reported thus far. In our study, we showed that the induction of adipogenic and osteogenic differentiation was successful in these four types of ADSCs. The results showed that lentiviral infection did not affect pluripotency (Figure S1).

### 3.2 The migration and Leydig‐like cell lineage differentiation ability of lentivirus‐infected ADSCs in vitro

**Figure 2:**
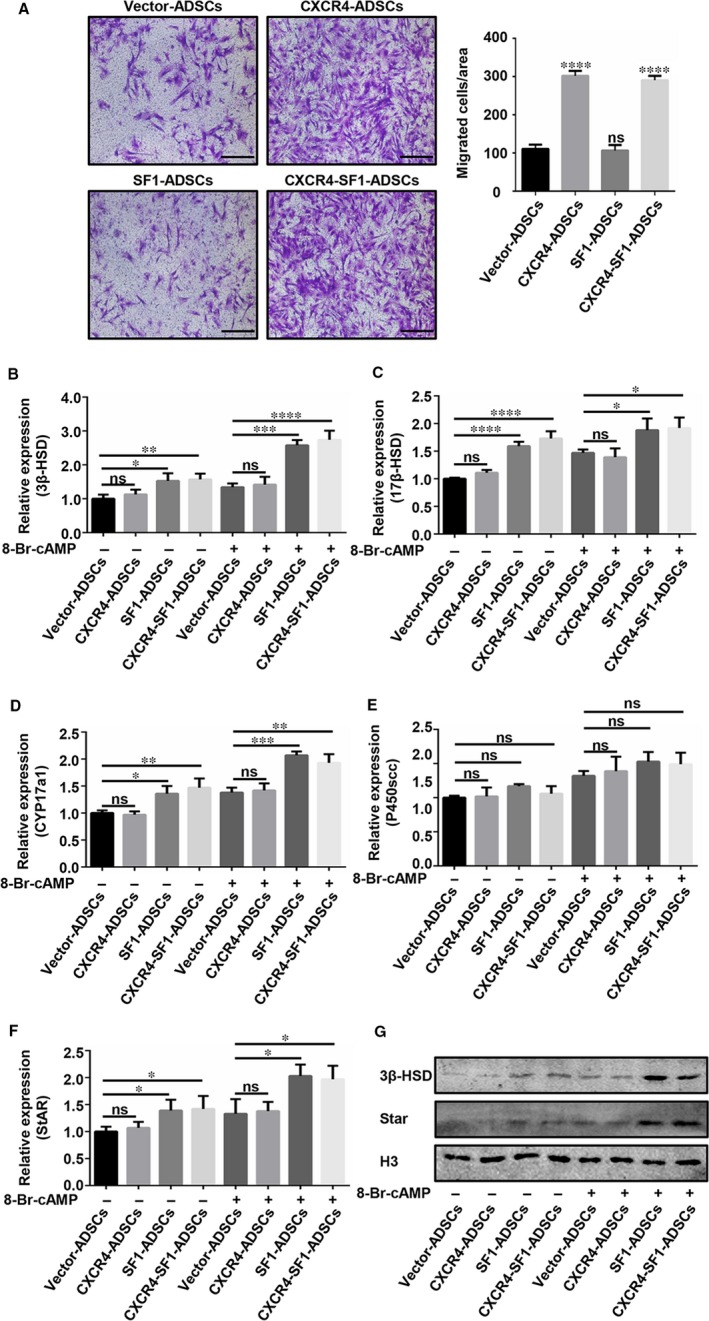
The migratory and Leydig‐like cell lineage differentiation abilities of lentivirus‐infected ADSCs. (A) A Transwell assay was used to detect the migratory ability of lentivirus‐infected ADSCs. Scale bar, 50 µm. The data from the Transwell assay are represented as the mean ± SD. The significance of differences was determined using one‐way ANOVA; ****P < .0001; NS, non‐significant difference; compared with the negative control. qPCR to detect the mRNA levels of 3β‐HSD (B), 17β‐HSD (C), CYP17a1 (D), P450scc (E) and StAR (F) and WB to detect the protein levels of 3β‐HSD and StAR after differentiation induction (G). *P < .05; **P < .01; ****P < .001; ****P < .0001; NS, non‐significant difference

After construction, we verified the migration capacities of the four cell types by a Transwell migration assay (Figure 2A). The migration capacities of CXCR4‐ADSCs and CXCR4‐SF1‐ADSCs were stronger than those of Vector‐ADSCs and SF1‐ADSCs (*P* < .0001). These results indicated that transfection of the CXCR4 gene could enhance the migratory ability of ADSCs in vitro.

8­Bromoadenosine 3′,5′­cyclic monophosphate (8­Br­cAMP) is a cyclic AMP‐dependent protein kinase (PKA) activator. 8‐Br‐cAMP, which functions as an inducer of ADSC differentiation into Leydig‐like cells, was applied to the four cell types, and fresh medium containing 8‐Br‐cAMP was used to replace half of the old medium every 2 days.^29^ Seven days later, the key protein (StAR) and enzymes (3β‐HSD, 17β‐HSD, CYP17a1 and P450scc) in testosterone synthesis were detected to determine the efficiency of differentiation induction. When 8‐Br‐cAMP was not added, the mRNA expression of 3β‐HSD, 17β‐HSD, CYP17a1 and StAR in SF1‐ADSCs and CXCR4‐SF1‐ADSCs was stronger than that in Vector‐ADSCs and CXCR4‐ADSCs. Furthermore, this phenomenon was also observed with 8‐Br‐cAMP treatment (Figure 2B‐E). The mRNA level of P450scc showed no differences among the four groups (Figure 2F). For further verification, we also detected 3β‐HSD, the most representative Leydig lineage‐specific marker,^30^, ^31^ and StAR, the protein involved in the rate‐limiting step of testosterone synthesis,^31^ at the protein level, and the results were consistent with those for the mRNA levels (Figure 2G). These results indicated that overexpression of the SF1 gene could promote the differentiation of ADSCs into Leydig‐like cells automatically.

### 3.3 CXCR4‐SF1‐ADSCs restored the damage to the testes caused by BPA

**Figure 3:**
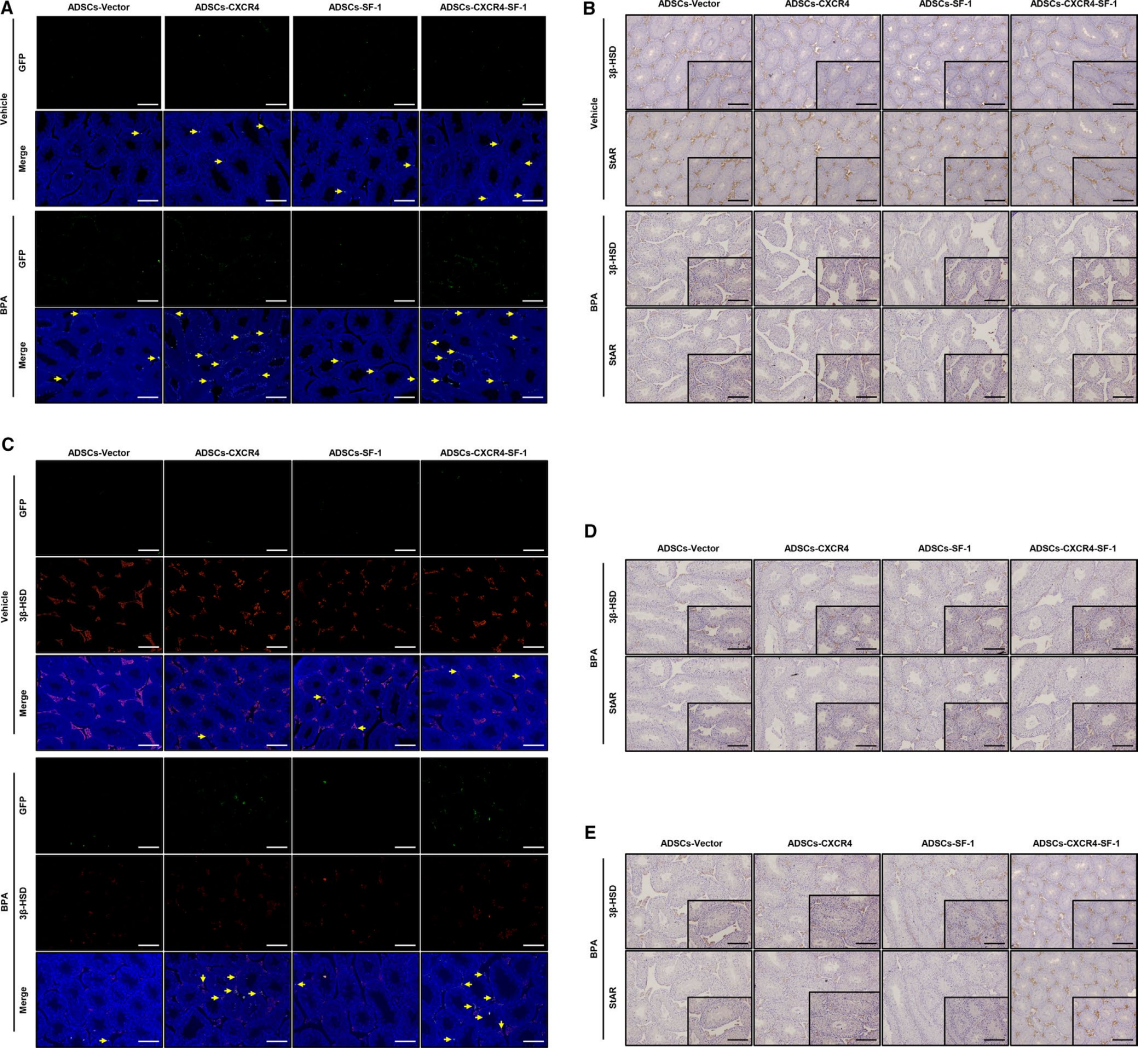
CXCR4‐SF1 bifunctional ADSCs repaired the damage to the testes caused by BPA. (A) One day after tail vein injection, immunofluorescence was used to verify the presence of migrated cells (GFP, green), marked by the yellow arrow, in the testes of mice treated with a vehicle or BPA. The nuclei were counterstained with DAPI (blue). Scale bar, 25 µm. One week (B), 3 wk (D) and 5 wk (E) after cell injection, the changes in the 3β HSD and StAR levels in the testes of the mice were evaluated by immunohistochemical analysis. Top scale bar, 25 µm; bottom scale bar, 50 µm. (C) Three weeks after injection of each of the four types of ADSCs, immunofluorescence was used to detect coexpression of 3β‐HSD and GFP (marked by the yellow arrow). GFP, green; 3β‐HSD, red; and DAPI, blue. Scale bar, 25 µm

After the four cell types and a BPA‐induced Leydig cell damage mouse model were successfully constructed (Figure S2), we inoculated the four types of ADSCs into mice treated with a vehicle or BPA via the tail vein (establishing 8 groups including Vehicle‐Vector‐ADSCs, Vehicle‐CXCR4‐ADSCs, Vehicle‐SF1‐ADSCs, Vehicle‐CXCR4‐SF1‐ADSCs, BPA‐Vector‐ADSCs, BPA‐CXCR4‐ADSCs, BPA + SF1‐ADSCs and BPA‐CXCR4‐SF1‐ADSCs). Twenty‐four hours after the tail vein injection, we harvested 5 mice in every group and detected the number of migrated cells in the testes by immunofluorescence staining of frozen sections. The migrated cells were identified by detecting green (GFP) fluorescence (Figure 3A). In the four control (vehicle‐treated) mouse groups, few migrated cells were found. In the BPA‐treated groups, the number of migrated cells was increased, and the number of migrated cells in the CXCR4‐ADSC and CXCR4‐SF1‐ADSC groups was significantly higher than that in the other two groups, which indicated that overexpression of the CXCR4 gene enhanced the migratory ability of ADSCs in vivo. In addition, we detected the expression of 3β‐HSD and StAR in the testes of mice in the different groups 1 week (Figure 3B), 3 weeks (Figure 3D) and 5 weeks (Figure 3E) after the cells were injected. We found that in the BPA‐treated mouse groups, the expression of 3β‐HSD and StAR in the testes increased as time progressed and reached normal levels at 5 weeks after CXCR4‐SF1‐ADSC inoculation compared with the same time after Vector‐ADSC inoculation; in contrast, cell inoculation did not affect the expression of 3β‐HSD or StAR in the vehicle‐treated mouse groups. This increased expression could also be observed in the BPA‐CXCR4‐ADSC and BPA‐SF1‐ADSC groups, but the increases were slight. Furthermore, the structural damage in the testicle was also restored 5 weeks after CXCR4‐SF1‐ADSC inoculation. These results suggested that CXCR4‐SF1‐ADSCs could localize to the testes and function as Leydig cells, thereby restoring structural and functional damage caused by BPA. To further confirm that this recovery phenomenon resulted from the migrated bifunctional ADSCs differentiating into Leydig‐like cells, we detected the colocalization of 3β‐HSD and GFP in testicular tissue by an immunofluorescence assay (Figure 3C). Coexpression of 3β‐HSD and GFP was found at 3 weeks after cell inoculation. Hardly any coexpression existed in the four control groups. However, in the four BPA‐treated groups, the coexpression was strongest in the CXCR4‐SF1‐ADSC group, followed by the CXCR4‐ADSC group, and weakest in the SF1‐ADSC and Vector‐ADSC groups. These results indicated that the cells that were positive for 3β‐HSD and GFP had differentiated from the inoculated CXCR4‐SF1‐ADSCs. In addition to the migratory ability, the Leydig cell lineage differentiation ability of the bifunctional ADSCs was also enhanced in vivo.

Considering that the ADSCs used were treated with a lentivirus, we evaluated morphological changes in the liver, lungs and kidneys as well as changes in liver and kidney function indexes in mice from every group after inoculation, and the results showed that the cells treated with the lentivirus had no toxic effects on the mice (Figure S3, Tables S2 and S3).

### 3.4 CXCR4‐SF1‐ADSCs restored the reproductive capacity of mice treated with BPA

**Figure 4:**
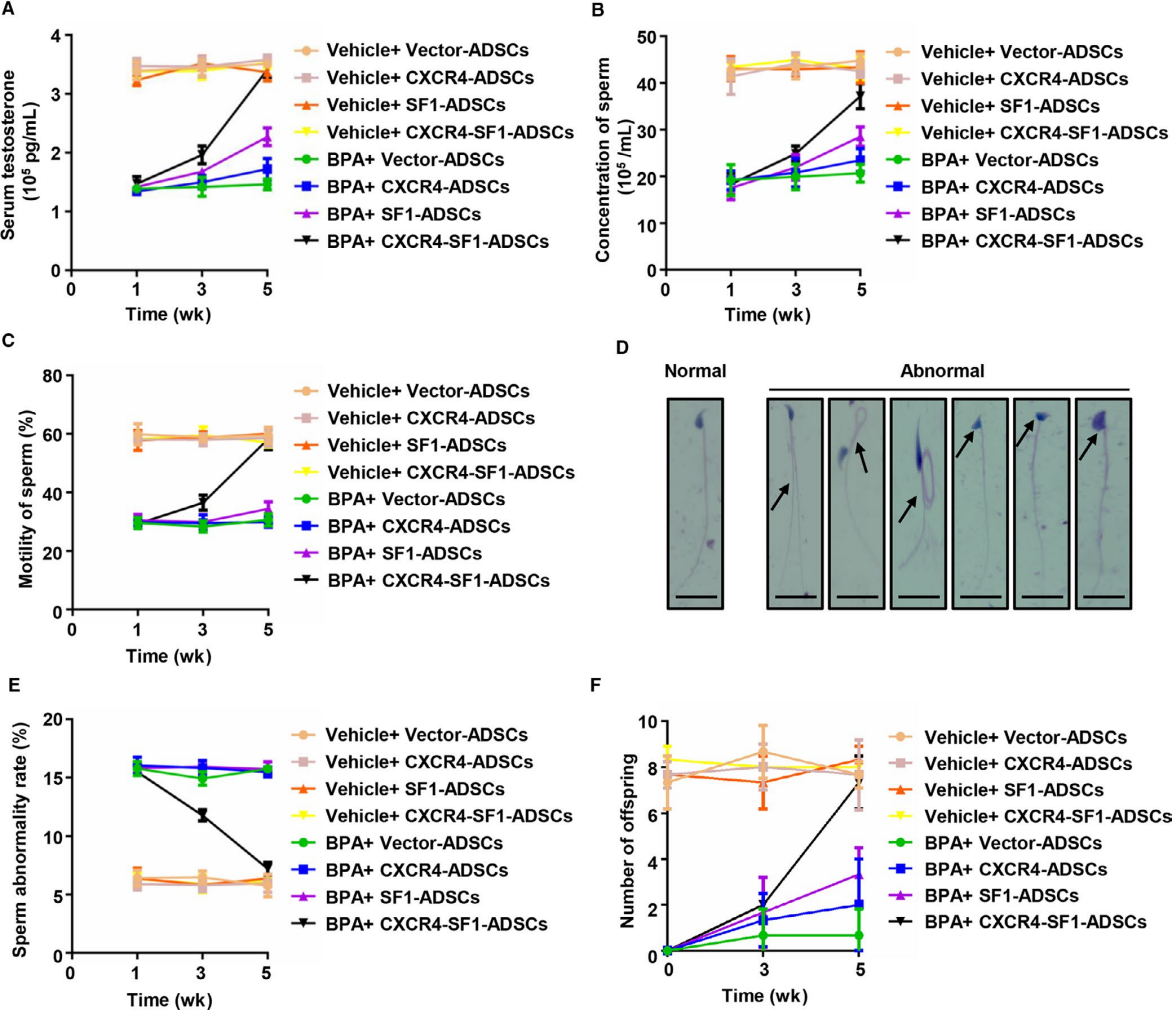
CXCR4‐SF1 bifunctional ADSCs repaired the reproductive capacity of mice treated with BPA. (A) After cell inoculation, ELISA analysis was applied to test serum testosterone changes in different groups over time. The changes in the sperm concentration (B), sperm motility (C), abnormal sperm rate (E) and offspring numbers (F) of the mice in different groups were monitored over time. (D) Haematoxylin and eosin staining was used to analyse the morphologies of normal and abnormal sperm. Scale bar, 10 μm

The ultimate purpose of this study was to rescue the reproductive dysfunction caused by Leydig cell dysfunction. The previous results proved that CXCR4‐SF1‐ADSCs could repair the testicular structure damaged by BPA. However, did these cells have a direct reparative effect on the reproductive system? Consequently, we measured the serum testosterone level (Figure 4A), sperm concentration (Figure 4B), sperm motility (Figure 4C), abnormal sperm rate (Figure 4E) and offspring numbers (Figure 4F) in different groups. Cell inoculation had no effects on these indexes in the vehicle‐treated mouse groups. However, all the indexes in the BPA‐CXCR4‐SF1‐ADSC group reached normal levels at 5 weeks after tail vein injection and compared with the indexes in the BPA‐Vector‐ADSC group, and those in the BPA‐CXCR4‐ADSC and BPA‐SF1‐ADSC groups showed slight recovery. Furthermore, Figure 4D shows the morphologies of normal and abnormal sperm (acrosome absence deformity, head deformity, tail rotation, double tail deformity, etc). These results demonstrated that CXCR4‐SF1‐ADSCs had a direct reparative effect on the reproductive capability of mice with Leydig cell dysfunction.

### 3.5 BSPRY might participate in the process of SF1‐mediated ADSC differentiation into Leydig‐like cells

**Figure 5:**
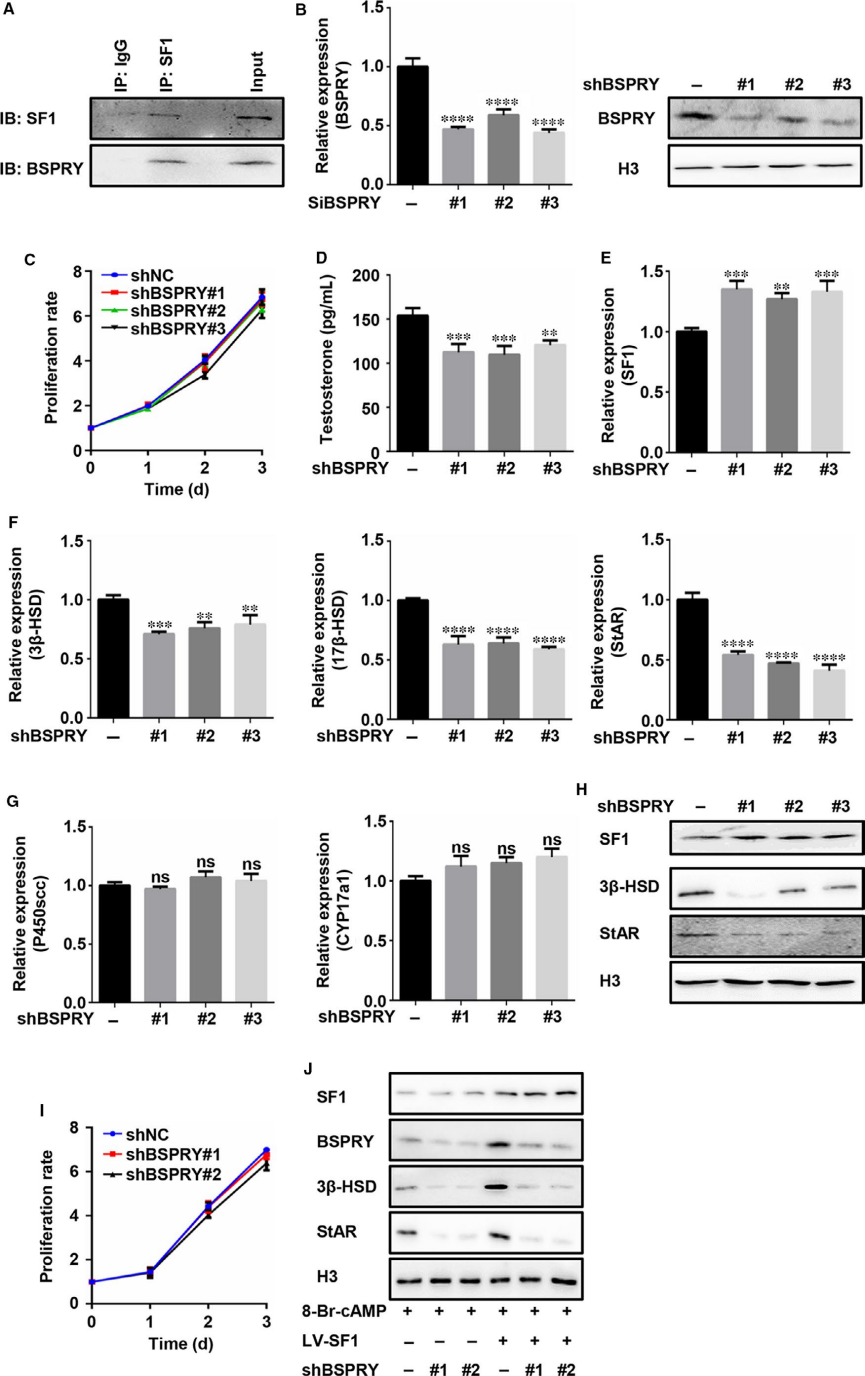
BSPRY might participate in the process of SF1‐mediated ADSC differentiation into Leydig‐like cells. (A) An immunoprecipitation assay was utilized to verify the relationship between SF1 and BSPRY. qPCR and Western blotting (B) were applied to detect the expression of BSPRY in TM3 cells transfected with shNC or shBSPRY #1/#2/#3. (C) The CCK8 method was used to detect the proliferation rates of TM3 cells transfected with shNC or shBSPRY #1/#2/#3. (D) An ELISA method was used to detect the testosterone concentration in culture supernatants of these cells. **P < .01, ****P < .001. (E, F, G) qPCR was used to determine SF1, 3β‐HSD, 17β HSD, StAR, P450scc and Cyp17a1 mRNA expression in TM3 cells treated with shNC or shBSPRY #1/#2/#3. (H) Western blotting was used to verify the SF1, 3β‐HSD and Star protein expression of the cells mentioned above. H3 is presented as a control. (I) The CCK8 method was used to detect the proliferation rates of ADSCs transfected with shNC or shBSPRY #1/#2. (J) Western blotting was used to detect the expression of SF1, BSPRY, 3β‐HSD and Star in ADSCs transfected with LV‐SF1 alone, shBSPRY #1/#2 alone or both vectors and then treated with 8‐Br‐cAmp

In previous experiments, we verified that CXCR4‐SF1‐ADSCs had the ability to home to injured testes and differentiate into Leydig‐like cells. We knew that SDF‐1/CXCR4 acted in the role of promoting the homing of the bifunctional ADSCs to the testes. However, which factors cooperate with SF1 during the differentiation process is still unknown. Baba Takashi et al^32^ carried out CHIP‐seq and RNA sequencing studies in Y‐1 cells. They reported that the mRNA levels of B‐box and SPRY Domain Containing Protein (BSPRY) decreased after interference with the SF1 gene. Moreover, BSPRY was highly expressed in testicular tissues and associated with the maintenance of mouse embryonic stem cell (ESC) pluripotency.^33^ To investigate the roles of BSPRY and SF1 in Leydig cell differentiation, we detected the interaction of SF1 and BSPRY by immunoprecipitation and found that the SF1 and BSPRY proteins exhibited direct binding (Figure 5A). TM3 cells (Leydig cells) were transfected with 3 siRNAs specific for BSPRY (shBSPRY #1/#2/#3), and the efficiencies of interference at the mRNA and protein levels were detected by qPCR and Western blotting, respectively (Figure 5B). The results showed that all 3 siRNAs worked well. The proliferation of TM3 cells was not affected by knocking down BSPRY expression (Figure 5C), but the level of testosterone produced by the TM3 cells decreased significantly (Figure 5D). Moreover, the mRNA levels of 3β‐HSD, 17β‐HSD and StAR (Figure 5F) were significantly decreased, but those of CYP17a1 and P450scc (Figure 5G) showed no significant changes in the TM3 cells with knocked down BSPRY expression. Interestingly, SF1 mRNA levels were slightly increased in these cells (Figure 5E). In addition, the changes in the 3 β‐HSD, StAR and SF1 protein levels (Figure 5H) agreed with their mRNA level changes. These results indicated that BSPRY might be involved in the SF1‐mediated testosterone synthesis process. These results were also confirmed in ADSCs. We transfected siRNAs (shBSPRY #1 and #2) into ADSCs, and the proliferation of the ADSCs was not influenced (Figure 5I). In addition, we transfected these siRNAs into Vector‐ADSCs or SF1‐ADSCs and cocultured these cells with 8‐Br‐cAMP. We found that interference with the BSPRY gene could offset the enhancement of Leydig‐like cell differentiation mediated by SF1 in the ADSCs (Figure 5J). This finding implies that BSPRY may assist SF‐1 in promoting the differentiation of ADSCs into Leydig‐like cells.

## 4 DISCUSSION

Currently, the clinical application of stem cell therapy has been conducted domestically and internationally. The associated achievements have provided treatment benefits for many diseases. Stem cell therapy has also been attempted in Leydig cell dysfunction‐related diseases. However, the low rates of cell migration and Leydig‐like cell differentiation after stem cell inoculation limit further research and applications of stem cell therapy in these diseases. In this study, we constructed a kind of bifunctional ADSCs that carried the CXCR4 gene, which encodes a stem cell homing‐related factor, and the SF1 gene, which encodes a Leydig cell embryonic differentiation‐ and development‐related factor. We found that CXCR4‐SF1‐ADSCs could migrate to the testes with dysfunctional Leydig cells and differentiate into Leydig‐like cells in vivo*.* Moreover, the testosterone level, sperm concentration, sperm motility, abnormal sperm rate and offspring number of BPA‐treated mice all returned to normal levels 5 weeks after CXCR4‐SF1‐ADSC inoculation. The bifunctional ADSCs perfectly solved migration and differentiation efficiency problems and might be considered a great therapeutic for Leydig cell dysfunction‐related diseases.

Leydig cell dysfunction, congenital or acquired, leads to defective synthesis and secretion of testosterone and then induces a series of androgen deficiency‐related diseases, such as sexual dysfunction, emotional disorder and cognitive impairment.^2^, ^30^ It is generally believed that stem cell transplantation therapy has advantages over hormone replacement therapy as this approach not only avoids side effects but also allows direct control of the hypothalamus‐pituitary axis similar to the control exerted by Leydig cells.^34^ Thus far, different kinds of stem cells have been reported to be able to differentiate into Leydig‐like cells in vitro.^35^, ^36^, ^37^ However, testosterone levels have shown only slight increases and have not returned to normal levels. Here, we found that following inoculation with CXCR4‐SF1‐ADSCs, testosterone levels in BPA‐treated mice recovered. Three weeks after inoculation, these cells were found in testicular tissues and were positive for 3β‐HSD, a Leydig lineage‐specific marker. This result indicated that the recovery of testosterone levels might be caused by the inoculated cells, which located to the testes, differentiated into Leydig‐like cells and then functioned as Leydig cells. However, 5 weeks later, the GFP fluorescence expressed by the CXCR4‐SF1‐ADSCs was too weak to be detected, so further work is needed to prove the function of the CXCR4‐SF1‐ADSCs. In addition, the structural damage in the testes caused by BPA also recovered following inoculation. We also evaluated testosterone levels in the supernatant of 8‐Br‐cAMP‐treated cells in vitro, but the expression of testosterone was not detected. This might be because during the inducement process, testosterone was diluted during culture medium changes and was therefore lower than the detection limit of the kit.

The cell inoculation method used for the treatment of Leydig cell dysfunction‐related diseases is also controversial. Chen Yong et al^12^ induced human ADSCs to differentiate into Leydig‐like cells in vitro and inoculated them into rats with Leydig cell damage. Although the testosterone levels in these rats increased, this direct testicular transplantation technique required an invasive operation, which might aggravate testicular injury, and the cell survival rate after transplantation could not be guaranteed. We chose to use a tail vein inoculation method in this study. The caudal vein cell inoculation technique is a simple and safe method that avoids aggravating testicular injury. Our results also confirmed that CXCR4‐SF1‐ADSCs inoculated via the caudal vein could migrate to testicular tissue with Leydig cell dysfunction and worked well. Therefore, this intravenous inoculation method has relatively great clinical application potential. Moreover, considering that all the ADSCs studied were treated with a lentivirus, we evaluated morphological changes in the liver, lungs and kidneys as well as changes in liver and kidney function indexes in mice in different groups after cell inoculation, and the results showed that the lentivirus‐treated cells had no toxic effects on the mice.

Various factors, such as dosage‐sensitive sex reversal gene 1 (DAX1) and anti‐Mullerian hormone gene (AMH), are involved in the SF‐1‐mediated differentiation, development and functional activities of Leydig cells.^38^, ^39^, ^40^ Here, we have proposed that a new gene, B‐box and SPRY Domain Containing Protein (BSPRY), may be involved in the SF1‐mediated differentiation and testosterone synthesis and secretion of Leydig cells. BSPRY is specifically highly expressed in testicular tissue, and Ikeda Mitsumi et al^33^ noted that BSPRY played a role in the maintenance of stem cell pluripotency. We proved that there was direct binding between SF1 and BSPRY by immunoprecipitation. Furthermore, after interfering with BSPRY, the protein expression of 3β‐HSD and StAR and the synthesis of testosterone in TM3 cells was decreased. In ADSCs, interference with BSPRY could inhibit the Leydig‐like cell differentiation ability enhanced by SF1. However, how BSPRY cooperates with SF1 to affect the function of Leydig cells needs to be further explored.

In conclusion, we successfully constructed CXCR4‐SF1‐ADSCs and inoculated them into BPA‐treated mice via the caudal vein. Additionally, we confirmed that the CXCR4‐SF1‐ADSCs could markedly increase testosterone levels in mice with Leydig cell dysfunction and restore the reproductive function of these mice. Thus, CXCR4‐SF1‐ADSCs have the potential to become a new therapeutic for treating Leydig cell dysfunction‐related diseases and are expected to be applied in clinical practice.

## CONFLICT OF INTEREST

The authors declare that there are no conflicts of interest.

## AUTHOR CONTRIBUTIONS

Li X and Xu A performed most of experiments and wrote the manuscript; Li X and Xu A analysed the data and prepared the Figures; Zhao G, Zhang Y, Yuan H and Guo Y performed the experiments; Lin P, Huang L, Li K, Zhang J and Li Q designed and guided all the experiments. All authors reviewed the manuscript. Lin P and Huang L revised the manuscript.

## Supporting information

Fig S1

Fig S2

Fig S3

Supplementary Material

## Data Availability

All data generated or analysed during this study are included in this article.
