# Supplementary figures and images for: CXCR4‐SF1 bifunctional adipose‐derived stem cells benefit for the treatment of Leydig cell dysfunction‐related diseases
Source: J Cell Mol Med. 2020 Mar 17;24(8):4633–45. doi: 10.1111/jcmm.15128 (PMC7176872; doi:10.1111/jcmm.15128)

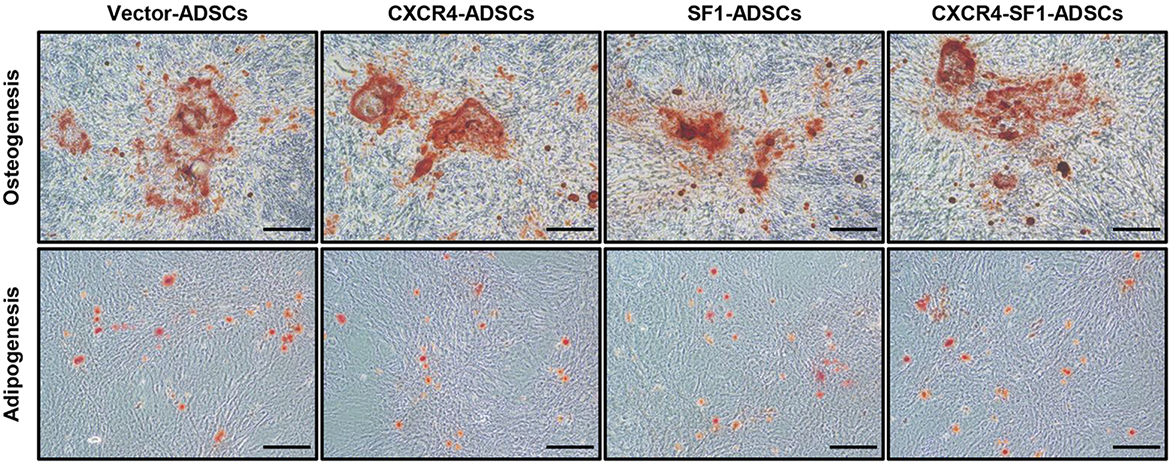

Supplement: Supplementary file 1 — Fig S1 [file JCMM-24-4633-s001.tif]

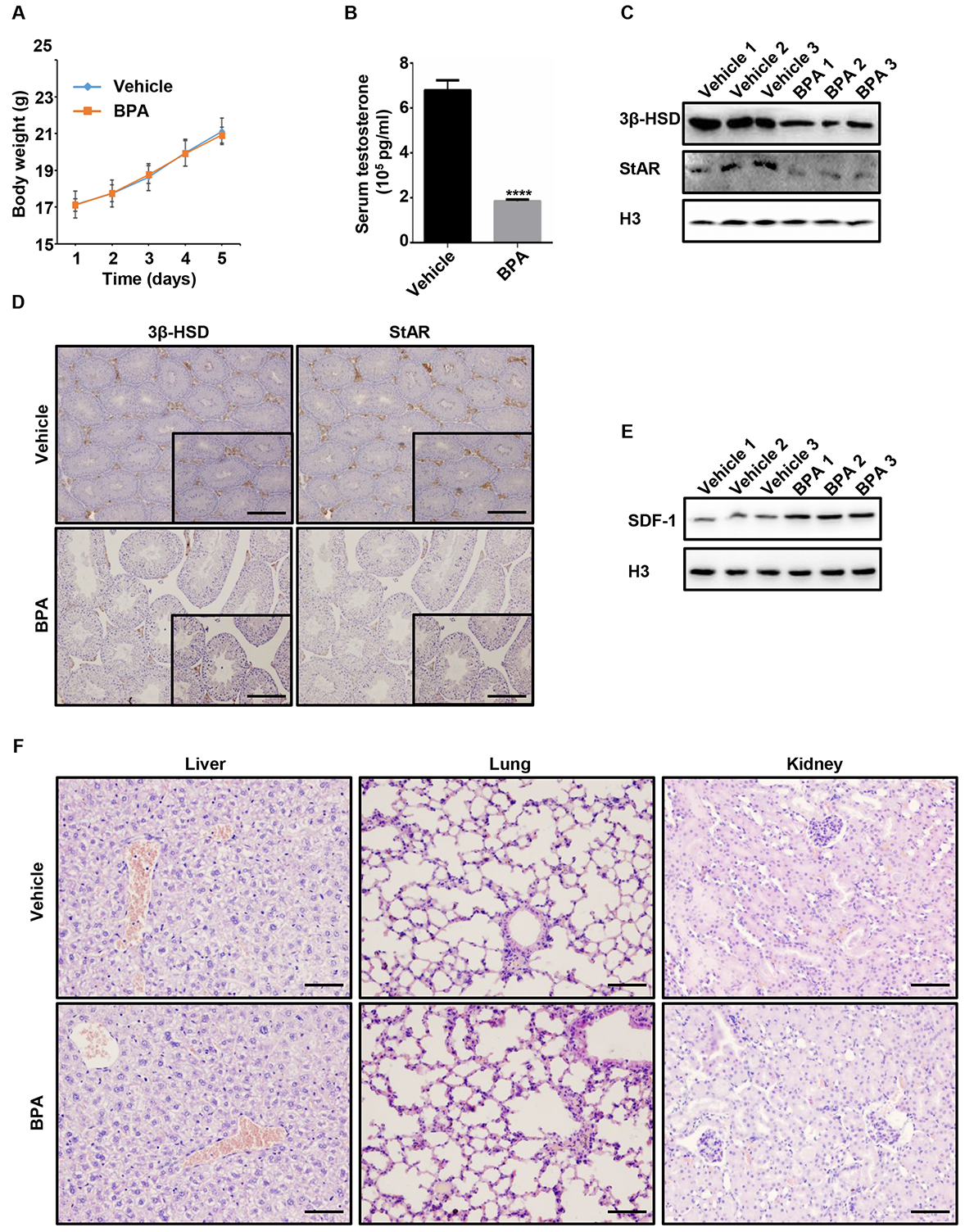

Supplement: Supplementary file 2 — Fig S2 [file JCMM-24-4633-s002.tif]

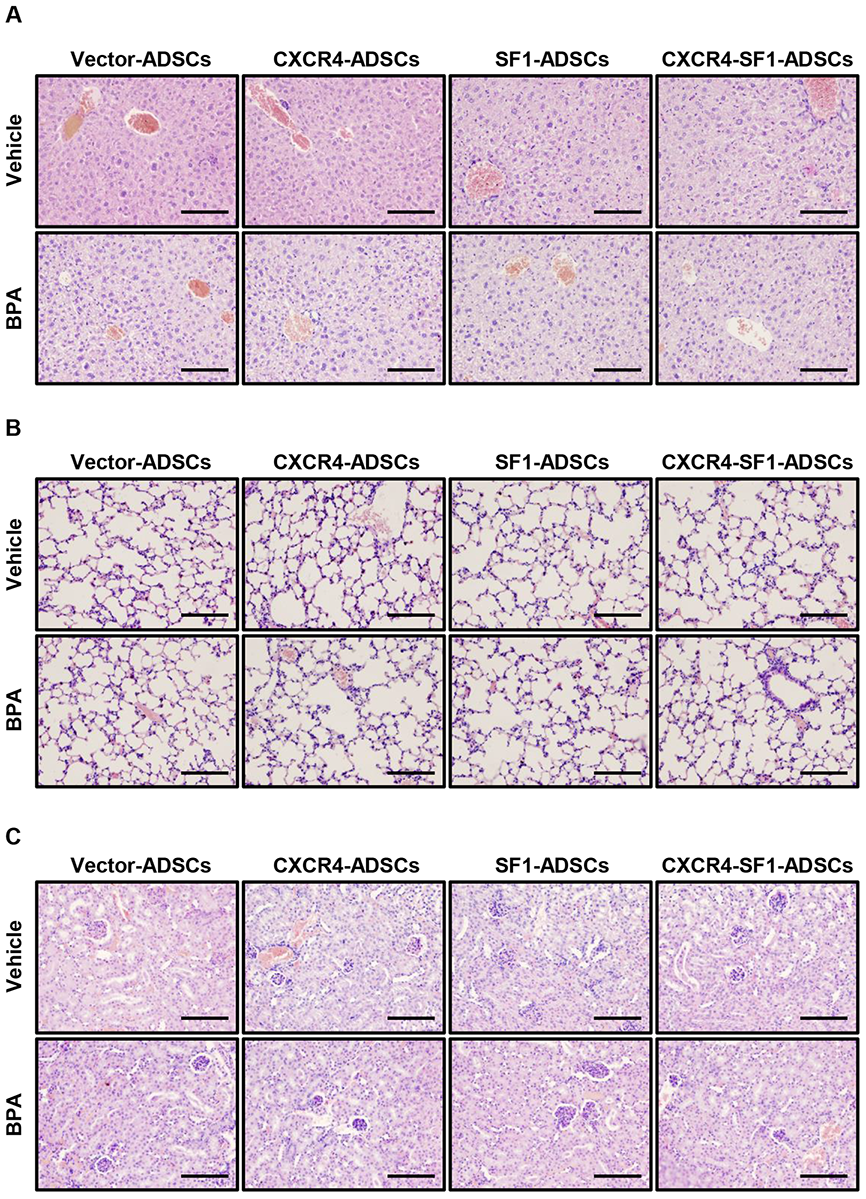

Supplement: Supplementary file 3 — Fig S3 [file JCMM-24-4633-s003.tif]
