# Supplementary material for: CXCR4‐SF1 bifunctional adipose‐derived stem cells benefit for the treatment of Leydig cell dysfunction‐related diseases
Source: J Cell Mol Med. 2020 Mar 17;24(8):4633–45. doi: 10.1111/jcmm.15128 (PMC7176872; doi:10.1111/jcmm.15128)
Supplement: Supplementary file 4 — Supplementary Material [file JCMM-24-4633-s004.doc]

**CXCR4-SF1 bifunctional adipose-derived stem cells benefit for the treatment of Leydig cell dysfunction-related diseases**

**Supplementary materials**

**Differentiation of lentivirus-infected ADSCs into adipogenic or osteogenic cells**

Four types of lentivirus-infected ADSCs were induced to undergo adipogenesis or osteogenesis (Fig. S1). The results showed that all of these ADSCs could differentiate into adipocytes and osteocytes, which indicated that lentiviral infection did not affect the pluripotency of ADSCs.

**BPA was used to establish a mouse model of Leydig cell damage**

To verify the function of CXCR4-SF1 bifunctional ADSCs *in vivo*, we established a BPA-mediated mouse model of Leydig cell damage by intraperitoneally injecting a vehicle or BPA (100 mg/kg for 5 days). Mouse body weight and food intake were determined. The data showed that BPA injection had no effect on mouse body weight (Fig. S2A). We measured the concentration of serum testosterone in mice by ELISA, and the level of testosterone decreased significantly after BPA treatment (Fig. S2B). In addition, the expression of the Leydig cell markers StAR and 3β HSD in the mouse testes was reduced in the BPA-treated group, as detected by immunohistochemical analysis and Western blotting (Fig. S2C and D). These data showed that the mouse model of Leydig cell damage was established successfully. The SDF-1 levels in the testes of mice treated with BPA were higher than those in the testes of mice treated with the vehicle (Fig. S2E). Furthermore, the use of BPA did not cause damage to the structures of the liver, lungs and kidneys (Fig. S2F), nor did it affect the functions of the liver and kidneys (P>0.05) in the treated mice (Table S1).

**Effects of lentivirus-infected ADSCs inoculation on liver and kidney functions of mice.**

Considering that all the ADSCs studied were treated with a lentivirus, we evaluated morphological changes in the liver, lungs and kidneys (Fig. S3A, B, and C) as well as changes in liver and kidney function indexes (Table S2 and S3) in mice from each group after inoculation, and the results showed that the lentivirus-treated cells had no toxic effects on the mice.

**Supplementary Tables**

**Table S1 The hepatic and renal functions indexes of the mice treated with Vehicle or BPA. (X±S)**

|  | **ALP (U/L)** | **ALT (U/L)** | **AST (U/L)** | **Urea (mmol/L)** | **Crea (μmol/L)** | **BUN (mg/dL)** |
| --- | --- | --- | --- | --- | --- | --- |
| **Vehicle** | **111.50±7.92** | **51.50±3.54** | **111.00±2.83** | **7.44±0.15** | **9.50±0.71** | **20.85±0.42** |
| **BPA** | **112.30±10.47** | **50.50±2.12** | **111.50±2.13** | **7.37±0.06** | **10.50±0.71** | **20.62±0.18** |

**Table S2 The hepatic functions indexes of the mice in different groups. (X±S)**

|  | | **ALP (U/L)** | **ALT (U/L)** | **AST (U/L)** |
| --- | --- | --- | --- | --- |
| **Vehicle** | **Vector-ADSCs** | **111.15±4.17** | **46.50±2.12** | **106.50±10.61** |
|  | **CXCR4-ADSCs** | **112.90±13.58** | **49.00±8.49** | **108.50±0.19** |
|  | **SF1-ADSCs** | **108.00±5.23** | **50.50±6.36** | **110.00±7.07** |
|  | **CXCR4-SF1-ADSCs** | **111.75±6.72** | **51.00±2.83** | **113.50±14.85** |
| **BPA** | **Vector-ADSCs** | **107.60±9.90** | **49.00±9.90** | **116.00±1.41** |
|  | **CXCR4-ADSCs** | **111.40±5.10** | **49.50±3.54** | **116.50±6.36** |
|  | **SF1-ADSCs** | **107.25±2.33** | **51.50±14.84** | **117.00±4.24** |
|  | **CXCR4-SF1-ADSCs** | **112.30±9.01** | **48.00±2.83** | **112.00±9.90** |

**Table S3 The renal functions indexes of the mice in different groups. (X±S)**

|  | | **Urea (mmol/L)** | **Crea (μmol/L)** | **BUN (mg/dL)** |
| --- | --- | --- | --- | --- |
| **Vehicle** | **Vector-ADSCs** | **7.07±0.46** | **9.00±1.41** | **19.78±1.29** |
|  | **CXCR4-ADSCs** | **6.83±0.81** | **9.50±0.71** | **19.12±2.26** |
|  | **SF1-ADSCs** | **7.04±0.43** | **9.50±0.71** | **19.70±1.21** |
|  | **CXCR4-SF1-ADSCs** | **7.59±0.36** | **10.00±1.41** | **21.24±1.01** |
| **BPA** | **Vector-ADSCs** | **7.59±0.41** | **10.00±0.00** | **21.25±1.15** |
|  | **CXCR4-ADSCs** | **7.71±0.29** | **9.50±0.71** | **21.57±0.81** |
|  | **SF1-ADSCs** | **7.40±0.19** | **8.50±0.71** | **20.71±0.53** |
|  | **CXCR4-SF1-ADSCs** | **7.60±0.46** | **10.50±0.71** | **21.27±1.29** |

**Supplementary Figure Legends**

**Figure S1 The pluripotency of lentivirus-infected ADSCs.** The osteogenic (Alizarin Red S) and adipogenic (Oil red O) abilities of the four types of lentivirus-infected ADSCs. Scale bar, 25 µm.

**Figure S2 Establishment of the model of BPA-induced Leydig cell damage.** The body weight (A) and serum testosterone levels (B) of mice intraperitoneally injected with a vehicle or BPA (100 mg/kg) for 5 days. Western blotting (C) and immunohistochemical (D) methods to determine the StAR and 3β‑HSD levels in the testes of mice treated with the vehicle or BPA. Scale bars: top, 25 µm; bottom, 50 µm. (E) Western blotting to detect SDF-1 protein expression in testis homogenates from mice treated with the vehicle or BPA. H3 was used as a control. (F) Hematoxylin and eosin staining of liver, lung and kidney tissue samples from mice treated with the vehicle or BPA. Scale bar, 25 µm. (Table S1) Hepatic and renal function indexes of mice treated with the vehicle or BPA (n=3 in every group). All indexes are presented as the mean ± SEM.

**Figure S3 Effects of lentivirus-infected ADSC inoculation on the liver and kidney functions of mice.**

Hematoxylin and eosin staining of liver (A), lung (B) and kidney (C) tissue samples from mice in every group. (Table S2 and S3) Hepatic and renal function indexes of mice in different groups (n=3 per group). All indexes are presented as the mean ± SEM.
